# SCLpredT: Ab initio and homology-based prediction of subcellular localization by N-to-1 neural networks

**DOI:** 10.1186/2193-1801-2-502

**Published:** 2013-10-03

**Authors:** Alessandro Adelfio, Viola Volpato, Gianluca Pollastri

**Affiliations:** School of Computer Science and Informatics, University College Dublin, Belfield, Dublin 4 Ireland; Complex and Adaptive Systems Laboratory, University College Dublin, Belfield, Dublin 4 Ireland

## Abstract

**Abstract:**

The prediction of protein subcellular localization is a important step towards the prediction of protein function, and considerable effort has gone over the last decade into the development of computational predictors of protein localization. In this article we design a new predictor of protein subcellular localization, based on a Machine Learning model (N-to-1 Neural Networks) which we have recently developed. This system, in three versions specialised, respectively, on Plants, Fungi and Animals, has a rich output which incorporates the class “organelle” alongside cytoplasm, nucleus, mitochondria and extracellular, and, additionally, chloroplast in the case of Plants. We investigate the information gain of introducing additional inputs, including predicted secondary structure, and localization information from homologous sequences. To accommodate the latter we design a new algorithm which we present here for the first time. While we do not observe any improvement when including predicted secondary structure, we measure significant overall gains when adding homology information. The final predictor including homology information correctly predicts 74%, 79% and 60% of all proteins in the case of Fungi, Animals and Plants, respectively, and outperforms our previous, state-of-the-art predictor SCLpred, and the popular predictor BaCelLo. We also observe that the contribution of homology information becomes dominant over sequence information for sequence identity values exceeding 50% for Animals and Fungi, and 60% for Plants, confirming that subcellular localization is less conserved than structure.

SCLpredT is publicly available at http://distillf.ucd.ie/sclpredt/. Sequence- or template-based predictions can be obtained, and up to 32kbytes of input can be processed in a single submission.

## Background

As the number of known protein sequences keeps growing, the necessity for fast, reliable annotations for these proteins continues to be of great importance, and is likely to remain so for the foreseeable future. Annotations, in the form of structural or functional information, may derive from experimental and computational methods. As experimental methods are expensive, laborious and not always applicable, a substantial amount of work has been carried out, and is ongoing in the bioinformatics research community, for developing computational approaches able to automatically predict a cohort of different protein features and descriptors.

Protein function prediction, in particular, is one of the major challenges of bioinformatics. Being able to annotate protein functions fast and cheaply by computational means would produce a quantum leap in our knowledge of biology at a molecular level. Such knowledge, if accurate, might be effectively harnessed for knowledge discovery and, ultimately, medical therapy and drug design.

One of the steps on the path to annotating protein function is determining the localization of proteins inside the cell. The two problems are strongly related as in order to work together proteins have to be in the same location. Hence, knowing were a protein is normally found is a first indication of which proteins it may interact with and what its ultimate function may be. As protein localization may be used as a starting point in function prediction systems, the former problem may be considered a subtask and an integral part of the latter. In (Casadio et al. 2008) and (Mooney et al. 2011) overviews of subcellular localization techniques are provided and many of the best performing public predictors are benchmarked.

As in the case of protein structure prediction or protein function prediction, subcellular localization methods typically follow one of two different main approaches. The first one is a homology, or “template-based” approach. According to this approach, the subcellular localization annotation of a protein may be transferred from homologous proteins (“templates”) whose location is known, having been experimentally determined. Hence, performances of these methods are affected by the availability of one or more templates for each protein whose localization has to be predicted. In many cases templates may not be available, rendering this approach unapplicable. Moreover, as sorting of proteins to their final location is generally performed by very small parts of sequences, or “motifs”, global sequence similarity searches in protein databases may be useless or problematic at best. In fact, two proteins having a very high sequence identity may differ in those motifs that are responsible for their locations. Conversely, two sequences having remote similarity may share that portion on which their sorting relies.

The second “ab initio” or “sequence-based” approach aims to predict subcellular localization exploiting information contained in the sequence alone, leaving aside homology information. Machine learning techniques, such as Support Vector Machines, Neural Networks or Clustering techniques, are particularly suited to this class of methods. In many cases, e.g. TargetP (Emanuelsson et al. 2000) and Protein Prowler (Bodén and Hawkins 2005), sequences are scanned in portions and pattern recognition techniques are used to train the predictor to recognize and locate significant motifs. Additionally, or alternatively, sequences may be processed as a whole, extracting global features that are related to their location and elaborating those features. The latter is the case of BaCelLo (Pierloni et al. 2006), LOCtree (Nair and Rost 2005) and WoLF PSORT (Horton et al. 2007).

In (Mooney et al. 2011) we proposed a system for protein subcellular localization based on a novel neural network architecture. That system, called **SCLpred**, was tested and its performances were compared to the main state of the art subcellular localization predictors such as BaCelLo (Pierloni et al. 2006), LOCtree (Nair and Rost 2005), SherLoc (Shatkay et al. 2007), TargetP (Emanuelsson et al. 2000), Protein Prowler (Bodén and Hawkins 2005) and WoLF PSORT (Horton et al. 2007). SCLpred is novel in that it can potentially exploit both global knowledge about a sequence, and discover and harness information about local motifs. Similar neural network architectures also proved to perform well in the prediction of transmembrane beta-barrels (Savojardo et al. 2011), discovery of functional peptides (Mooney et al. 2012) and prediction of enzymatic classes (Volpato et al. 2013).

In this article we set out to achieve three distinct goals: gauging whether retraining SCLpred on larger, up-to-date datasets yields gains over our previous architecture; incorporating into the SCLpred architecture homology information in the form of experimental subcellular localization annotations; enhancing the input encoding, to include predicted structural information.

Although SCLpred, which is based on a new neural network model, performs well (significantly better than its competitors, in the benchmarks in (Mooney et al. 2011)), it contains a larger number of free parameters than most of the alternatives, as it takes very large motifs (up to several tens of residues) from a protein sequence as inputs. Because of this, it has plenty of spare capacity to accommodate a large training set, which densely samples the space of proteins. We thus built a new dataset from the Gene Ontology (Consortium 2000), a project that aims to unify language for annotation producing a structured, dynamic and controlled vocabulary for describing genes and their roles in any organism. The GO contains three independent ontologies structured as directed acyclic graphs in which each node is a “GO term” and has well-known and well-defined connections with its parents and with its children. As of 16/01/2012 there were 108,938,299 GO annotations to 12,833,146 distinct proteins assigned by 36 different databases that have subscribed to the project. We relied on GO to build an enhanced dataset of proteins annotated for their subcellular localizations, which are reported in the “cellular component” ontology of GO.

Subcellular localizations can be predicted at different levels of granularity, and subcellular localization predictors classify proteins into anywhere between 3-4 and more than 10 classes. In SCLpred we adopted 4 classes for Fungi and Animals and 5 for Plants. Here, given we rely on larger datasets, we expand the number of classes by one in all three kingdoms, by including the “organelle” class. We also make a change by replacing the “secreted” class with proteins annotated as belonging to the extracellular region. This class contains most proteins annotated as secreted and aligns our class definition to works such as (Nair and Rost 2005), (Shatkay et al. 2007) and (Horton et al. 2007). The two classes are strongly related because proteins annotated as “secretion by cell” usually carry out their function in the extracellular region.

Another direction of investigation we explore is incorporating into the input to SCLpred richer information than the primary sequence and multiple sequence alignments. In particular, we consider two kinds of additional information: predicted secondary structure for each sequence; localization of sequences homologous to each sequence in our dataset. We test the effect of this information on SCLpred performances separately, but to handle the second type of input, some material changes to the SCLpred predictive architecture had to be made. In the remainder of this article we will thus refer to the former architecture (essentially the same as in the original SCLpred (Mooney et al. 2011)) as “sequence-based”, and to the latter including homology information as “template-based”.

## Results and discussion

In this work we present results we achieved on the same sets of proteins, using the three different kinds of input coding and the two different predictive architectures described in the methods section. We compare, for each taxonomic group, results achieved using the sequence-based model on MSA_dataset (only sequence information) and on MSA+SS_dataset (sequence and secondary structure information) and using the template-based model on MSA+HOM_dataset (sequence, plus homology information from functional “templates”). For all comparisons we use the indices described in (Mooney et al. 2011). In particular specificity (Spec), sensitivity (Sens), false positive rate (FPR) and Matthews correlation coefficient (MCC) measure performances for each class. To measure global performances we use generalized correlation (GC) and the percentage of correct predictions (Q).

The results obtained in the first case are useful as an element of comparison as the architecture and the information provided to the model are essentially the same of (Mooney et al. 2011). This allows us to gauge the impact of an expanded dataset and of the introduction of an additional output class (“organelle”).

For all the three taxonomic groups we observe similar trends that are shown in Table 1. It can be seen how the template-based architecture performs generally better than the sequence-based one, tested on both MSA_dataset and MSA+SS_dataset. In the Fungi case we register a GC increase of 6% for the MSA+HOM case compared to the sequence-based architecture on MSA_dataset and of 10% compared to sequence-based on MSA+SS_dataset, with standard deviations of 1.1-1.5%. In the Animal case we observe GC improvements of, respectively, 14% and 15% (standard deviations 0.4-0.5%). In Plants GC is 1% and 5% higher (standard deviations 1%). Performances based on the Q index follow the same trend: 74% against 73% and 71% in the Fungi case (deviations 1.1%); 79% against 70% and 68% in the Animal case (deviations 0.3%); 60% against 58% and 56% in the Plant case (deviations 0.5%).

**Table 1:** **10-fold cross validation results**

				Sequence-based models						Template-based model		
		**MSA**				**MSA+SS**				**MSA+HOM**		
	**Spec**	**Sens**	**MCC**	**FPR**	**Spec**	**Sens**	**MCC**	**FPR**	**Spec**	**Sens**	**MCC**	**FPR**
						**Fungi**						
cyto	0.38	0.20	0.22	0.04	0.34	0.24	0.23	0.05	0.39	0.46	0.35	0.08
extr	0.50	0.57	0.52	0.01	0.49	0.49	0.48	0.01	0.51	0.73	0.59	0.01
mito	0.73	0.61	0.60	0.05	0.72	0.60	0.59	0.05	0.72	0.68	0.63	0.06
nucl	0.75	0.88	0.57	0.32	0.75	0.84	0.54	0.31	0.83	0.82	0.62	0.19
orga	0.76	0.70	0.67	0.05	0.73	0.69	0.65	0.06	0.77	0.71	0.68	0.05
GC	0.56	(0.018)			0.52				**0.62**	(0.011)		
Q	0.73	(0.004)			0.71				**0.74**	(0.005)		
						**Animals**						
cyto	0.44	0.42	0.35	0.08	0.40	0.43	0.33	0.09	0.55	0.67	0.55	0.07
extr	0.73	0.78	0.72	0.04	0.70	0.78	0.70	0.04	0.76	0.87	0.78	0.04
mito	0.72	0.71	0.66	0.05	0.70	0.70	0.65	0.05	0.79	0.81	0.76	0.04
nucl	0.78	0.79	0.60	0.19	0.79	0.76	0.59	0.17	0.91	0.81	0.76	0.06
orga	0.62	0.58	0.54	0.06	0.58	0.57	0.50	0.07	0.73	0.73	0.68	0.04
GC	0.60	(0.005)			0.59				**0.74**	(0.004)		
Q	0.70	(0.004)			0.68				**0.79**	(0.003)		
						**Plants**						
chlo	0.68	0.63	0.55	0.10	0.66	0.62	0.53	0.10	0.69	0.62	0.55	0.09
cyto	0.53	0.54	0.40	0.13	0.47	0.49	0.33	0.16	0.54	0.54	0.41	0.13
extr	0.41	0.45	0.41	0.02	0.42	0.34	0.36	0.01	0.41	0.49	0.43	0.02
mito	0.40	0.37	0.32	0.06	0.39	0.31	0.28	0.05	0.39	0.39	0.32	0.07
nucl	0.60	0.67	0.51	0.14	0.59	0.64	0.49	0.13	0.67	0.69	0.58	0.11
orga	0.65	0.59	0.54	0.07	0.59	0.62	0.51	0.09	0.61	0.62	0.53	0.09
GC	0.49	(0.008)			0.45				**0.50**	(0.011)		
Q	0.58	(0.005)			0.56				**0.60**	(0.004)		

Results on Fungi, Animal and Plant by all three architectures (MSA, MSA+SS and MSA+HOM) measured in 10-fold cross-validation on the reduced_dataset (see text for details). Best results are in bold. Standard deviations for MSA and MSA+HOM global results are in brackets.

These results show how introducing homology-based information is generally advantageous. On the other hand we observe that adding secondary structure information does not improve the performances of the system. It is not entirely clear why this is the case, although the noisy (predicted) nature of secondary structure predictions is likely to have a role. It is also possible that the introduction of secondary structure composition may bias the training process and prevent the networks from learning subtler information from the sequence and MSA. This hypothesis is confirmed by the shorter training times we observe when secondary structure in provided as an input.

In Table 2 we show the performances of our system compared to SCLpred and BaCelLo (Pierloni et al. 2006). All systems are assessed by 10-fold cross-validation on the same BaCelLo set. In order to test our system on the BaCelLo set, we retrained it excluding from MSA_dataset and MSA+HOM_dataset (separately for each fold) all the proteins with a sequence identity of 30% or greater to any sequence in BaCelLo set. Moreover we excluded from our sets the class organelle that was considered neither in BaCelLo nor in SCLpred.

**Table 2:** **Results on the BaCelLo sets**

	SCLpred		BaCelLo		MSA		MSA+HOM	
				**Fungi**				
	**Spec**	**Sens**	**Spec**	**Sens**	**Spec**	**Sens**	**Spec**	**Sens**
cyto	0.46	0.39	0.39	0.60	0.74	0.30	0.72	0.34
mito	0.72	0.78	0.72	0.81	0.79	0.87	0.75	0.92
nucl	0.83	0.82	0.85	0.67	0.80	0.94	0.83	0.94
secr/extr	0.86	0.85	0.85	0.94	0.99	0.72	0.99	0.62
GC	0.67		0.66		**0.69**	(0.01)	0.68	(0.014)
Q	0.75		0.70		0.80	(0.008)	**0.81**	(0.007)
				**Animals**				
	Spec	Sens	Spec	Sens	Spec	Sens	Spec	Sens
cyto	0.58	0.54	0.41	0.65	0.66	0.33	0.72	0.45
mito	0.77	0.74	0.66	0.76	0.68	0.93	0.69	0.98
nucl	0.83	0.85	0.85	0.65	0.77	0.92	0.83	0.92
secr/extr	0.93	0.93	0.91	0.91	0.97	0.86	0.95	0.91
GC	0.72		0.67		0.76	(0.007)	**0.81**	(0.006)
Q	0.82		0.74		0.80	(0.006)	**0.84**	(0.006)
				**Plants**				
	Spec	Sens	Spec	Sens	Spec	Sens	Spec	Sens
chlo	0.68	0.79	0.76	0.73	0.83	0.56	0.82	0.61
cyto	0.39	0.36	0.47	0.52	0.40	0.76	0.47	0.78
mito	0.49	0.34	0.54	0.51	0.56	0.50	0.60	0.55
nucl	0.83	0.76	0.76	0.72	0.64	0.77	0.73	0.90
secr/extr	0.89	0.85	0.65	0.85	0.67	0.40	0.76	0.41
GC	0.63		0.59		0.57	(0.022)	**0.66**	(0.019)
Q	**0.68**		**0.68**		0.62	(0.021)	0.67	(0.019)

Results on Fungi, Animal and Plant by the MSA and MSA+HOM architectures compared against BaCelLo, measured in 10-fold cross-validation on the BaCelLo sets (see text for details). SCLpred and BaCelLo results are from (Mooney et al. 2011), Table 3. Best results are in bold. Standard deviations for MSA and MSA+HOM global results are in brackets.

The new system performs generally better for Fungi (69% GC by the MSA version and 81% Q of the MSA+HOM version are the best of the four systems). The MSA+HOM version of our system is the most accurate on the Animal set (81% GC and 84% Q). For plant set, the 66% GC of the MSA+HOM version is the best result achieved among the four systems, while the 67% Q of the same version is slightly below the 68% Q of BaCelLo and SCLPred. Given the small size of the sets, standard deviations for SCLpred in this case are close to 2% for Plants for both Q and GC, 1% for Fungi, and 0.6% for Animal.

### Effect of sequence identity to best template

**Figure 1:**
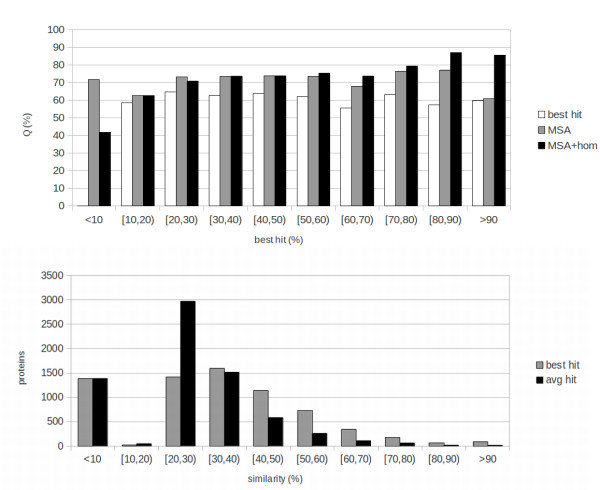
**Fungi: results and template distribution.** **Top:** results of the 10-fold cross-validation tests for Fungi. Results are presented in bins corresponding to the sequence identity of the examples to the best template that could be found. Black bars represent template-based results (MSA-hom), grey bars sequence-based results (MSA) and white bars baseline results obtained by assigning a protein to the class having the highest value in the template profile (input to the homology-based part of the network). **Bottom:** number of proteins with templates within given ranges of sequence identity for Fungi. Black bins represent average sequence identity, grey bin identity to the best template.

**Figure 2:**
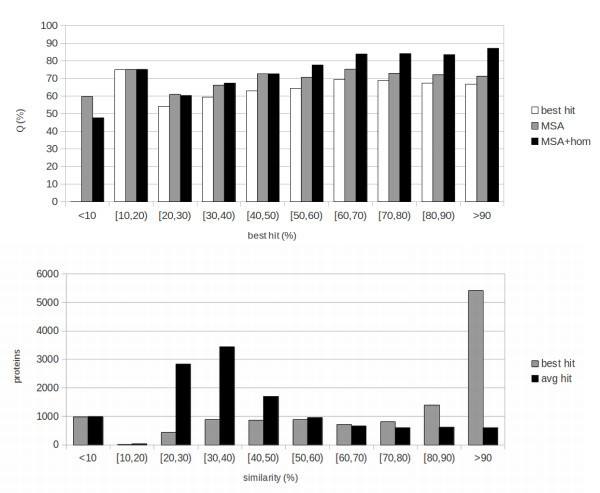
**Animals: results and template distribution.** **Top:** results of the 10-fold cross-validation tests for Animals. Results are presented in bins corresponding to the sequence identity of the examples to the best template that could be found. Black bars represent template-based results (MSA-hom), grey bars sequence-based results (MSA) and white bars baseline results obtained by assigning a protein to the class having the highest value in the template profile (input to the homology-based part of the network). **Bottom:** number of proteins with templates within given ranges of sequence identity for Animals. Black bins represent average sequence identity, grey bin identity to the best template.

**Figure 3:**
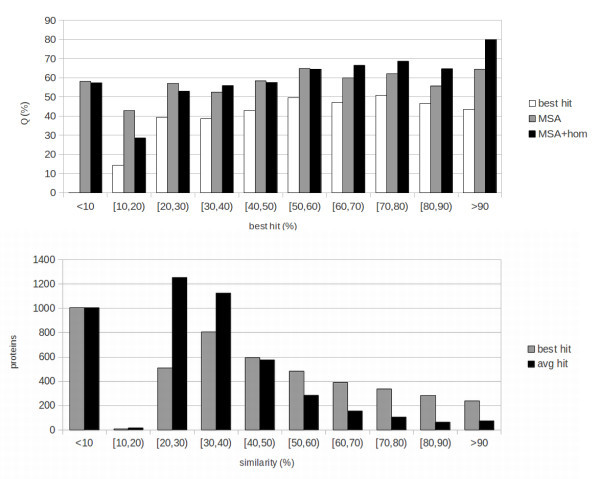
**Plant: results and template distribution.** **Top:** results of the 10-fold cross-validation tests for Plants. Results are presented in bins corresponding to the sequence identity of the examples to the best template that could be found. Black bars represent template-based results (MSA-hom), grey bars sequence-based results (MSA) and white bars baseline results obtained by assigning a protein to the class having the highest value in the template profile (input to the homology-based part of the network). **Bottom:** number of proteins with templates within given ranges of sequence identity for Plants. Black bins represent average sequence identity, grey bin identity to the best template.

In order to measure the effect of sequence identity to the best template (that is: how much the quality of templates affects the performances of the system) we subdivide the results into bins of 10% sequence identity. The results are reported at the top of Figures 1, 2 and 3 for, respectively, Fungi, Animals and Plants. We compute three different sets of results in this binned version: results including homology (MSA+HOM) reported as black bars; sequence-based results (MSA, grey bars); baseline results obtained by simply assigning a protein to the class that has the highest value in the weighted average of templates (homology-based part of the input), reported as white bars.

**Table 3:** **Full_dataset**

Class	GO term	Fungi	Animal	Plant
Chloroplast	“chloroplast”			1870
Cytoplasm	“cytosol”	2648	4533	1887
Extracellular	“extracellular region”	238	4104	293
Mitochondrion	“mitochondrion”	1607	5014	715
Nucleus	“nucleus”	4440	15201	1653
	“vacuole”			
Organelle	“endoplasmic reticulum”	1720	5429	1526
	“perexisome”			
	“golgi apparatus”			
Total		10653	34281	7944

Number of protein sequences per class for each taxonomic group, extracted from GO using GO terms (full_dataset).

The numbers of proteins in different classes of sequence identity to the best template are reported at the bottom of Figures 1, 2 and 3. These distributions should be taken into account while evaluating aggregate results in Table 1. For instance, while in the Animal case a sizeable fraction of instances has very high quality templates, this is not the case for Plants and, especially, for Fungi. Because of this the Animal set is “easier” for the template-based predictor, while the Plant and Fungi sets are more challenging.

Consistent with the fact that thresholds for transferring function tend to be somewhat higher than those for transferring structure, the MSA+HOM results are superior to the MSA ones (sequence-based) for values of sequence identity to the best template above 50% for Fungi and Animals, and for 60% for Plants. This is roughly consistent with, e.g. (Rost et al. 2003), where subcellular localization is shown to transfer very poorly by homology for sequence identities below 40%, followed by a sharp increase for higher identities. By contrast, structure is known to transfer by homology for much lower levels of identity (e.g. see (Baker and Sali 2001)). Some structural features have been shown to be conserved for, and especially be predictable based upon, even remote homology at sequence identity levels between 10% and 20% (Mooney and Pollastri 2009). It should also be noted how here we are only measuring where a purely sequence-based predictor is outperformed by a template-based one, and not where homology is informative for inferring subcellular localization. Our experiments suggest that homology is no more informative than the sequence below a certain level of sequence identity, but we cannot conclude that it is not informative. To elucidate this, direct studies relying on sequence comparison as in (Rost et al. 2003) are more appropriate. Below the 50-60% threshold MSA and MSA+HOM results are similar, with the exception of very low levels of sequence identity (under 10% for Fungi and Animals, and 20% for Plants) where the MSA results are superior. Although the MSA+HOM systems have, alongside the template profile, the same inputs as the MSA ones, for the vast majority of examples the MSA+HOM systems are trained to rely upon the template profile for the prediction. For this reason, they learn predominantly on the sequence-based part of the input only in a narrow range of examples (those where the sequence carries more information than the templates). As such, the sequence-based part of MSA+HOM systems is effectively trained on a smaller set than in the MSA case, and it is not entirely surprising that they are outperformed by this more specialized set of sequence-based predictors when good templates are absent.

In all cases both MSA and MSA+HOM systems perform better than the baseline. This shows how, for subcellular localization, template information is not as robust as in the case of structural prediction, and needs to be complemented with information about motifs and composition extracted from the sequence, and also how N-to-1 NN are effective at extracting this information, and at merging different sources in the MSA+HOM case.

## Conclusions

Protein subcellular localization prediction is very closely related to the goal of protein function prediction: knowing in which cellular component proteins carry out their function is a first indication of what their function may be. Hence, protein localization may be used as starting point for function prediction systems.

In this paper we explored three different directions of investigation for enhancing SCLpred, the subcellular localization predictor presented in (Mooney et al. 2011). First, we widened the sequence dataset used to train our predictor, relying on the GO project, and we expanded the number of predictable classes by one, including the “organelle” class. Second, we incorporated into each sequence of our dataset global homology information from proteins of known localization. In order to handle this additional information, we designed a new neural network model, which we are here presenting for the first time. Third, we enhanced the input encoding to include predicted structural information.

For each of these directions, we ran separate tests and showed that the best results are achieved by introducing homology information from proteins of known subcellular localization. The contribution of homology to the predictive ability of our system was assessed at different levels of sequence identity. The new system was compared with the previous version of SCLpred as well as with BaCelLo (Pierloni et al. 2006) on the same sequence dataset, achieving state of the art results.

In our future research we intend to investigate other kinds of structural information at amino acid level. Features such as relative solvent accessibility, contact density (Vullo et al. 2006a), structural motifs (Mooney et al. 2006) and intrinsic disorder (Vullo et al. 2006b; Walsh et al. 2011) are all predictable by various public programs, including the public server Distill (Baú et al. 2006) developed in our laboratory. The contribution of these features to the model prediction performances may be assessed separately and in conjunction, in order to select the most informative data to exploit. Moreover, experiments on a larger number of classes will be carried out in order to evaluate our system response. Prediction of motifs involved in protein localization will be also attempted exploiting N1-NN model. By highlighting those motifs that significantly contribute to the feature vector and by rejecting those that contribute minimally, it should be possible to draw up sets of the motifs that are strongly related to protein localization.

SCLpredT is freely available for academic users at http://distillf.ucd.ie/sclpredt/. The server is free for academic users and implements both sequence-based and template-based predictions. Up to 32kbytes of text, corresponding to approximately 100 average-sized proteins, can be processed in a single submission.

## Materials and methods

### Datasets

The datasets used to train and test our system are built using QuickGO, a web-based tool designed for browsing the GO database. We started from the GO release of 06/06/2011 and built three datasets, one for each kindgom considered. We selected proteins annotated with GO terms related to the subcellular localizations we consider.

Table 3 shows, for each class, the GO term or terms used to filter sequences, and the number of proteins obtained per class for each taxonomic group. Constructing this set, called **full_dataset**, proteins annotated as “inferred from electronic annotation”, “non-traceable author statement”, “no data” and “inferred by curator” were left out in order to exclude sequences of little-known origin or of uncertain localization.

From the full_dataset we excluded sequences shorter than 30 amino acids. Then we reduced redundancy, separately for each taxonomic group, performing an all-against-all BLAST (Altschul et al. 1997) search with an e-value of 10^-3^ and excluding sequences with a sequence identity of 30% or greater to any other sequence in the group that was retained. Table 4 reports the final numbers of sequences in the sets after redundancy reduction(**reduced_dataset**). We use **reduced_dataset** for training/testing purposes.

**Table 4:** **Reduced_dataset**

	Fungi	Animal	Plant
chlo			1143
cyto	678	1500	1013
mito	1247	1978	451
nucl	3661	5694	1101
orga	1274	1778	845
extr	95	1457	224
total	6955	12407	4777

Number of sequences in the final train/test set (reduced_dataset).

#### ***Input coding***

As in (Mooney et al. 2011), we enrich the description of protein sequences using residue frequency profiles from alignments of multiple homologous sequences (MSA). This is common practice in many predictive systems of structural and functional properties of proteins, as MSA provide information about the evolution of a protein (Rost and Sander 1993). We built a “profile” for each protein in the following way: the *k*-th residue in a protein is encoded as a sequence of 20 real numbers in which each number is the frequency of one of the 20 amino acids in the *k*-th column of the MSA, gaps excluded; an additional 21st real number is used to represent the frequency of gaps in the *k*-th column. Sequence alignments are extracted from uniref90 (Suzek et al. 2007) from February 2010 containing 6,464,895 sequences. The alignments are generated by three runs of PSI-BLAST (Altschul et al. 1997) with parameters b = 3000 (maximum number of hits) and e = 10^-3^ (expectation of a random hit) (Mooney et al. 2011). We refer to this first encoding as **MSA_dataset**.

In a second step, we encode proteins in our dataset adding three inputs per residue describing the secondary structure that the residue is predicted to belong to, according to the Porter server (Pollastri and McLysaght 2005; Pollastri et al. 2007). We call this encoding **MSA+SS_dataset**.

We train and test two versions of the sequence-based architecture using, respectively, the **MSA_dataset** and the **MSA+SS_dataset**, which contain the same proteins, but have different input encoding.

In another set of experiments we add homology information from proteins of known subcellular localization. Similarly to (Pollastri et al. 2007; Mooney 2009; Walsh et al. 2009a; Walsh et al. 2009b), homology is used as a further input to the predictor, alongside a measure of its estimated quality. The predictor itself determines how to weigh the information coming directly from the input sequence and MSA, and how to weigh the annotations coming from homologous proteins into the final prediction. Homology information itself is extracted by performing a BLAST (Altschul et al. 1997) search for each sequence in reduced_dataset against the full_dataset with an e-value of 10^-3^. For each sequence *i* in reduced_dataset we select the *K*_*i*_ sequences in full_dataset having an Identity Score higher than 30% (but smaller than 95%, to exclude the protein itself) and we calculate a vector *N*+1 terms long, where *N* is the number of classes predicted (five in Fungi and Animal cases, six in the Plant case) as:1

where  is a vector of *N* units in which the *k*-th entry is set to one if the *j*-th protein belongs to the *k*-th class, to zero otherwise; *I*_*ij*_ is the identity between sequence *i* in the reduced_dataset and sequence *j* among the *K*_*i*_ in full_dataset that is homologous to sequence *i*. Taking the cube of the identity scores reduces the contribution of low-similarity proteins while high-similarity sequences are available. The *N*+1-th element in the vector *T*_*i*_ measures the significance of the information stored in the vector and is computed as the average identity, weighed by the cubed identity. That is:2

In other words: each protein in reduced_dataset is aligned against full_dataset; the significant hits (templates) are retrieved, with their (known) subcellular locations; a profile of subcellular locations is compiled from these templates, where templates that are more closely related to the protein are weighed more than more remote ones, according to the score in Equation 2; this subcellular location profile is provided as an extra input to the network. Notice that in the case in which all templates from full_dataset are in the same subcellular location class, vector  has only two non-zero components: the entry corresponding to the class (which in this case is 1), and the last entry which measures the average sequence identity of the templates.

Thus, in this third set of experiments (**MSA+HOM_dataset**), a vector containing homology information is associated to each sequence+MSA. Again, while the proteins are the same as in the **MSA_dataset** and the **MSA+SS_dataset**, the information provided to the predictor is different.

### Predictive architecture

In this work we test two different predictive systems based on the model proposed in (Mooney et al. 2011). This model is a N-to-1 neural network, or N1-NN, composed by a sequence of two two-layered feed-forward neural networks.

The first architecture we test is essentially the same as in (Mooney et al. 2011), in which different numbers of inputs per residue are fed to the system. In N1-NN a lower level network  takes as input a window or motif of a fixed number of residues. 21 (MSA_dataset case) or 24 (MSA+SS_dataset) real numbers encode each residue. The lower level network is replicated for each of the (overlapping) motifs in the sequence and produces a vector of real numbers as output. A feature vector  for the whole sequence is calculated as the sum of the output vectors of all the lower network replicas (Mooney et al. 2011).  contains a sequence of descriptors automatically learned in order to minimize the overall error, that is, to obtain an optimal final prediction. Thus  can be thought of as a property-driven adaptive compression of the sequence into a fixed number of descriptors. The vector  is obtained as:3

where  is the sequence of real numbers (21 or 24) associated with the residue *i* in a *L*-length sequence, *k* is a normalization constant (set to 0.01 in all our tests) and *c* is a constant that determines the length of the window of residues (2*c*+1) that is fed to the network. We use *c*=20 in all the experiments in this article, corresponding to motifs of 41 residues. We obtained a value of *c*=20 from preliminary tests, in which it proved (marginally) better than 10 and 15, but we also considered that the average size for motifs that sort a protein to a subcellular location is generally smaller (but close to) 40 residues. For instance the average length of signal peptides in eukaryotes is approximately 20 residues (Bendtsen et al. 2004), and 35-40 is an upper size bound for most known signals and NLS (Bendtsen et al. 2004; Cokol et al. 2000). We set *k*=0.01 because the number of replicas of  is typically between several tens and a few hundreds. Different choices for *k* are possible in principle, including making it a learnable parameter, although we have not explored this option.

The feature vector  is fed to a second level network that performs the final prediction as:4

**Figure 4:**
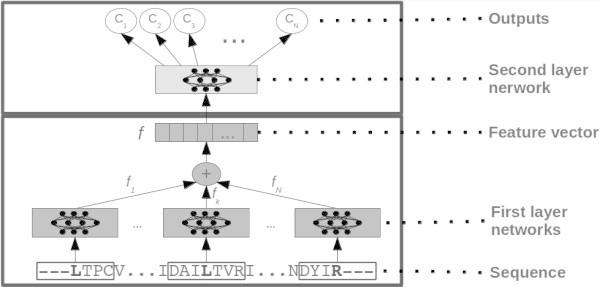
**A standard N-to-1 Neural Network, as used in the sequence-based experiments in this article and in (Mooney et al.** 2011 **).** All motifs from the sequence are input to replicas of input-to-feature neural networks (grey boxes at the bottom of the image), the feature vectors output are added up, to produce a global feature vector (*f*). *f* is then mapped to the property of interest through a feature-to-output neural network (light grey box at the top of the image).

A standard N-to-1 NN is depicted in Figure 4.

In the second (template-based) architecture we add a second lower level neural network, that takes as input the additional vector *T* included in the MSA+HOM_dataset. So the feature vector *f* is now calculated as5

in which  and  are two-layer perceptrons as in the standard N1-NN. Hence  is now composed of two parts: one that contains information relating to the sequence, MSA, and secondary structure when present; a second part that contains information about annotations extracted from homologous proteins. Both parts are automatically learned, and the compound vector is mapped into the property of interest through a two-layer perceptron  as in the standard N1-NN.

The overall number of free parameters in the second architecture can be calculated as:6

in which *I* is the number of inputs for the network  depending on the input coding and on the context window chosen,  is the number of hidden units in the network , *F*_1_ is the number of descriptors in the first part in of the feature vector, *T* is the number of inputs in vector ,  is the number of the hidden units in the network , *F*_2_ is the number of descriptors in the second part in of the feature vector,  is the number of the hidden units in the network  and *O* is the number of the classes being predicted.

Hence the parameters that control the size of the model are , *F*_1_, , *F*_2_ and .

**Figure 5:**
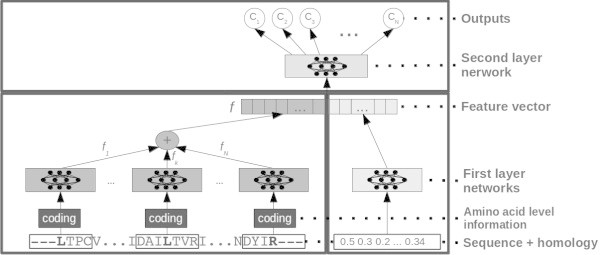
**A modified N-to-1 Neural Network, as used in the template-based experiments in this article.** The left side is essentially the same as the standard N-to-1 Neural Network depicted in Figure 4. A further, single neural network maps homology information into a number of descriptors that are concatenated to the descriptors obtained from the sequence. In this case the feature vector *f* contains two parts, one sequence-based, one homology-based. The top feature-to-output network is essentialy the same as the standard N-to-1 NN case.

A modified N-to-1 NN is depicted in Figure 5.

#### ***Training***

We perform tests on three kingdoms (Fungi, Animal and Plant) and with three different architectures (**MSA_dataset**,**MSA+SS_dataset** and **MSA+HOM_dataset**), or nine tests in total. Each test is run in 10-fold cross validation. For each fold a different tenth of the overall dataset is reserved for testing, while the remaining nine tenths are used for learning the parameters of the N1-NN. In particular these nine tenths are further split into a proper training part (eight tenths of the total), and a validation set (one tenth of the total) which is used to monitor the training process but not for learning the N1-NN parameters by gradient descent. For each fold we repeat the training 3 times, with 3 different training/validation splits. Thus for each of the 9 kingdom/architecture combinations we have 3 repetitions x 10 folds, or 30 separate N1-NN training runs in total. In each training set, sequences are replicated as necessary in order to obtain classes of roughly the same size.

Training is performed by gradient descent on the error, which is modelled as the relative entropy between the target class and the output of the network. The overall output of the network (output layer of *N*^(*o*)^()) is implemented as a softmax function, while all internal squashing functions in the networks in both models are implemented as hyperbolic tangents. The examples are shuffled between epochs. We use a momentum term of 0.9 that speeds up overall training times by a factor 3-5 compared to no momentum. The learning rate is kept fixed at 0.2 throughout training.

Parameters for both the first and the second architecture were experimentally determined in preliminary tests. For the sequence-based N-to-1 NN architecture we use *N*^*H*^ = 14, *F* = 12 and . For the template-based architecture that includes homology we set , *F*_1_ = 10, , *F*_2_ = 6 and . These values result in approximately 12,500 free parameters for the sequence-based N-to-1 NN, and just over 10,000 for the template-based one. Each training is carried out for up to 10 days on a single state of the art core. Performance on the validation set is measured every ten training epochs, and the ten best performing models on validation are stored. For each fold we ensemble average the three best models saved (one for each repetition) and evaluate them on the corresponding test set. The final result for the 10-fold cross-validation is the average of the results over the ten test sets.

## References

[CR1] Altschul S, Madden T, Schäffer A, Zhang J, Zhang Z, Miller W, Lipman D (1997). Gapped BLAST and PSI-BLAST: a new generation of protein database search programs. Nucleic Acids Res.

[CR2] Baker D, Sali A (2001). Protein structure prediction and structural genomics. Science.

[CR3] Baú D, Martin A, Mooney C, Vullo A, Walsh I, Pollastri G (2006). Distill a suite of web servers for the prediction of one-, two-and three-dimensional structural features of proteins. BMC Bioinformatics.

[CR4] Bendtsen J, Nielsen H, Von Heijne G, Brunak S (2004). Improved prediction of signal peptides: SignalP 3.0. J Mol Biol.

[CR5] Bodén M, Hawkins J (2005). Prediction of subcellular localization using sequence-biased recurrent networks. Bioinformatics.

[CR6] Casadio R, Martelli P, Pierleoni A (2008). The prediction of protein subcellular localization from sequence: a shortcut to functional genome annotation. Brief Funct Genomic Proteomic.

[CR7] Cokol M, Nair R, Rost B (2000). Finding nuclear localization signals. EMBO Rep.

[CR8] Consortium TGO (2000). Gene ontology: tool for the unification of biology. Nat Genet.

[CR9] Emanuelsson O, Nielsen H, Brunak S, Von Heijne G (2000). Predicting subcellular localization of proteins based on their N-terminal amino acid sequence. J Mol Biol.

[CR10] Horton P, Park K, Obayashi T, Fujita N, Harada H, Adams-Collier C, Naka K (2007). WoLF PSORT:protein localization predictor. Nucleic Acids Res.

[CR11] Mooney C, Pollastri G (2009). Beyond the twilight zone: automated prediction of structural properties of proteins by recursive neural networks and remote homology information. Proteins.

[CR12] Mooney C, Vullo A, Pollastri G (2006). Protein structural motif prediction in multidimensional ø-ψ space leads to improved secondary structure prediction. J Comput Biol.

[CR13] Mooney C, Wang YH, Pollastri G (2011). SCLpred: protein subcellular localization prediction by N-to-1 neural networks. Bioinformatics.

[CR14] Mooney C, Haslam N, Pollastri G, Shields DC (2012). Towards the improved discovery and design of functional Peptides: common features of diverse classes permit generalized prediction of bioactivity. PLoS ONE.

[CR15] Nair R, Rost B (2005). Mimicking cellular sorting improves prediction of subcellular localization. J Mol Biol.

[CR16] Pierloni A, Martelli PL, Fariselli P, Casadio R (2006). BaCelLo: a balanced subcellular localization predictor. Bioinformatics.

[CR17] Pollastri G, McLysaght A (2005). Porter: a new, accurate server for protein secondary structure prediction. Bioinformatics.

[CR18] Pollastri G, Martin AJ, Mooney C, Vullo A (2007). Accurate prediction of protein secondary structure and solvent accessibility by consensus combiners of sequence and structure information. BMC Bioinformatics.

[CR19] Rost B, Sander C (1993). Improved prediction of protein secondary structure by use of sequence profiles and neural networks. Proc Nutl Acad Sci USA.

[CR20] Rost B, Liu J, Nair R, Wrzeszczynski K, Ofran Y (2003). Automatic prediction of protein function. Cell Mol Life Sci.

[CR21] Savojardo C, Fariselli P, Casadio R (2011). Improving the detection of transmembrane β-barrel chains with N-to-1 extreme learning machines. Bioinformatics.

[CR22] Shatkay H, Höglund A, Brady S, Blum T, Dönnes P, Kohlbacher O (2007). Sherloc: high-accuracy prediction of protein subcellular localization by integrating text and protein sequence data. Bioinformatics.

[CR23] Suzek BE, Huang H, McGarvey P, Mazumder R, Wu CH (2007). UniRef: comprehensive and non-redundant UniProt reference clusters. Bioinformatics.

[CR24] Volpato V, Adelfio A, Pollastri G (2013). Accurate prediction of protein enzymatic class by N-to-1 Neural Networks. BMC Bioinformatics.

[CR25] Vullo A, Walsh I, Pollastri G (2006a). A two-stage approach for improved prediction of residue contact maps. BMC Bioinformatics.

[CR26] Vullo A, Bortolami O, Pollastri G, Tosatto S (2006b). Spritz: a server for the prediction of intrinsically disordered regions in protein sequences using kernel machines. Nucleic Acids Res.

[CR27] Walsh I, Baù D, Martin A, Mooney C, Vullo A, Pollastri G (2009a). Ab initio and template-based prediction of multi-class distance maps by two-dimensional recursive neural networks. BMC Struc Biol.

[CR28] Walsh I, Martin A, Mooney C, Rubagotti E, Vullo A, Pollastri G (2009b). Ab initio and homology based prediction of protein domains by recursive neural networks. BMC Bioinformatics.

[CR29] Walsh I, Martin A, Di Domenico T, Vullo A, Pollastri G, Tosatto S (2011). CSpritz: accurate prediction of protein disorder segments with annotation for homology, secondary structure and linear motifs. Nucleic Acids Res.

